# Assessment of axillary temperature for the evaluation of normal body temperature of healthy young adults at rest in a thermoneutral environment

**DOI:** 10.1186/s40101-017-0133-y

**Published:** 2017-02-22

**Authors:** Shuri Marui, Ayaka Misawa, Yuki Tanaka, Kei Nagashima

**Affiliations:** 1 Body Temperature and Fluid Laboratory (Laboratory of Integrative Physiology), Faculty of Human Sciences, Waseda University, Mikajima 2-579-15, Tokorozawa, Saitama 359-1192 Japan; 2 Institute of Applied Brain Sciences, Waseda University, Tokorozawa, Saitama 359-1192 Japan

**Keywords:** Core temperature, Regular body temperature, Tympanic temperature, Digital thermometer, Infrared thermometry, Menstrual cycle, Body mass index, Prediction measurement, Thermal sensation, Healthy people

## Abstract

**Background:**

The aims of this study were to (1) evaluate whether recently introduced methods of measuring axillary temperature are reliable, (2) examine if individuals know their baseline body temperature based on an actual measurement, and (3) assess the factors affecting axillary temperature and reevaluate the meaning of the axillary temperature.

**Methods:**

Subjects were healthy young men and women (*n* = 76 and *n* = 65, respectively). Three measurements were obtained: (1) axillary temperature using a digital thermometer in a predictive mode requiring 10 s (*T*_ax-10 s_), (2) axillary temperature using a digital thermometer in a standard mode requiring 10 min (*T*_ax-10 min_), and (3) tympanic membrane temperature continuously measured by infrared thermometry (*T*_ty_). The subjects answered questions about eating and exercise habits, sleep and menstrual cycles, and thermoregulation and reported what they believed their regular body temperature to be (*T*_reg_).

**Results:**

*T*_reg_, *T*_ax-10 s_, *T*_ax-10 min_, and *T*_ty_ were 36.2 ± 0.4, 36.4 ± 0.5, 36.5 ± 0.4, and 36.8 ± 0.3 °C (mean ± SD), respectively. There were correlations between *T*_ty_ and *T*_ax-10 min_, *T*_ty_ and *T*_ax-10 s_, and *T*_ax-10 min_ and *T*_ax-10 s_ (*r* = .62, *r* = .46, and *r* = .59, respectively, *P* < .001), but not between *T*_reg_ and *T*_ax-10 s_ (*r* = .11, *P* = .20). A lower *T*_ax-10 s_ was associated with smaller body mass indices and irregular menstrual cycles.

**Conclusions:**

Modern devices for measuring axillary temperature may have changed the range of body temperature that is recognized as normal. Core body temperature variations estimated by tympanic measurements were smaller than those estimated by axillary measurements. This variation of axillary temperature may be due to changes in the measurement methods introduced by modern devices and techniques. However, axillary temperature values correlated well with those of tympanic measurements, suggesting that the technique may reliably report an individual’s state of health. It is important for individuals to know their baseline axillary temperature to evaluate subsequent temperature measurements as normal or abnormal. Moreover, axillary temperature variations may, in part, reflect fat mass and changes due to the menstrual cycle.

## Background

In most animals, body temperature is an important determinant for metabolism, movement, and neural activity [1–3]. Homeothermic animals, in particular, maintain a constant body temperature using various autonomic and behavioral processes [4]. However, the meaning of the term body temperature is sometimes vague. In large animals, including human beings, the body temperature represents the temperatures of two separated physical compartments: core and shell [5], and reports indicate that thermal inputs from both the core body and the skin activate thermoregulatory responses [6, 7].

We propose that the core body temperature is used as a surrogate for the body temperature in clinical medicine, and accurate monitoring involves placement of a thermometer such as a thermistor probe or thermocouple in the core body, e.g., the rectum or esophagus [8, 9]. More practical methods such as thermometry in the oral cavity, axilla, and ear canal are used in clinics and at home as the first step in the evaluation of infection, inflammation, and medication effects. These methods aim to assess the core temperature although the temperatures measured are those of the body shell. Among them, axillary temperature measurement has been widely used to evaluate patient temperature for years [9–11], probably due to the ease of axillary access [10]. However, the influence of the environmental temperature and incorrect placement of the thermometer lead to erroneous body temperature measurement [9]. Additionally, some more recently introduced digital thermometers, while capable of producing rapid results, utilize a predictive algorithm that could augment measurement errors and tend to show lower values [12–14].

“Normal body temperature” was defined as the axillary temperature measured using a mercury thermometer (approximately 37.0 °C) [15]. However, axillary temperature varies among people, and temperatures ranging from 36.2 to 37.5 °C are accepted as normal [15, 16]. This range may compensate for various factors that influence measurement. The factors include measurement errors and environment temperature. Moreover, we speculate that the wide range of axillary temperature reflects physical and physiological characteristics affecting the shell temperature, such as fat mass, skin blood flow, or basal metabolic rate. The existence of human temperature variation indicates that a comparison of an individual’s temperature with the normal range may not accurately evaluate their state of health. Instead, it is more important to compare the individual’s current temperature with their personal baseline temperature. For example, we can identify a fever based on a temperature that is 0.5 °C greater than the personal normal temperature.

In the present study, we aimed to reevaluate the meaning of the normal body temperature determined by measurements of axillary temperature. Previous studies assessed the importance of axillary temperature measurements by comparing them to core temperature measurements [11–13, 17–24]. However, these studies were limited to small groups, patients, and newborns. Therefore, we first compared the axillary and tympanic temperatures of over 100 healthy subjects of a similar age in the same thermoneutral environment and during the same season. Tympanic temperature was utilized as a surrogate for core temperature [25, 26]. We also compared each subject’s perceived personal baseline body temperature with the axillary temperature we recorded. Finally, we tested our hypothesis that axillary temperature deviations are related to physical, physiological, and behavioral characteristics.

## Methods

### Subjects

Healthy college students were recruited for the study (76 males and 65 females, aged 20.7 ± 1.6 and 20.7 ± 1.9 years (mean ± SD), respectively). Experiments were conducted from August to October (autumn in Japan). Body temperature was measured from 2:00 to 3:00 p.m. in an experimental room maintained at an ambient temperature of 25.4 ± 2.0 °C and relative humidity of 62 ± 9% (mean ± SD, respectively). All subjects were instructed to wear light clothing (such as T-shirts and long pants), and no subjects reported discomfort during the study. Written informed consent was obtained from all individual participants prior to commencing the study. The Human Research Ethics Committee of the Faculty of Human Sciences of Waseda University approved all the procedures. The study was also conducted in accordance with the Declaration of Helsinki.

Subjects were instructed to avoid exercise the day before the experiment and eschew food intake for 1 h before arriving at the experimental room. In addition, we verified that subjects wore lighter clothes. While sitting in a chair for at least 30 min, the subjects completed a questionnaire (12 questions for males and 13 for females) about sleeping and eating habits, menstrual cycle (females), exercise, and body temperature. The questionnaire included a question asking each participant to state his or her own regular body temperature (*T*_reg_).

Axillary temperatures were determined with a digital thermistor probe (MC612, Omron Healthcare, Inc., Kyoto, Japan). The accuracy and resolving power of the thermistor sensor are ±0.1 and 0.1 °C, respectively. The measurement was performed twice, using different modes provided in the probe. One temperature was obtained using the standard mode: subjects were asked to place the thermometer in their axilla until the temperature displayed was stable, which usually took 10 min (*T*_ax-10 min_). The other was assessed using the predictive mode: subjects were instructed to place the thermometer in the same manner, and the value was determined by an algorithm (based on the immediate increase in temperature that occurs when the subject places the instrument) within 10 s (*T*_ax-10 s_). Subjects conducted these measurements by themselves after instruction by a researcher.

After the measurement of *T*_ax-10 min_ and *T*_ax-10 s_, the subject’s tympanic membrane temperature (*T*_ty_) was monitored with an infrared sensor probe (CE Thermo, NIPRO Corp., Osaka, Japan) as a surrogate for estimating core temperature [25, 26]. The sensor probe was placed in the left ear canal with the assistance of a researcher. The probe occluded the ear canal, and the researcher adjusted the placement of the probe so as to make the sensor show the highest value (i.e., ideal direction of the probe). The data was recorded at 30-s intervals and stored on a computer, till when the value became stable (±0.1 °C for 3 min, usually took 10–15 min).

Body mass index was calculated as weight (kg)/height^2^ (m^2^) and classified as follows: underweight, 18.4 kg/m^2^ or below; normal weight, 18.5–24.9 kg/m^2^; overweight, 25.0 kg/m^2^ or above [27].

### Statistics

We drew histograms demonstrating the grouped *T*_reg_, *T*_ax-10 s_, *T*_ax-10 min_, and *T*_ty_ data. The data for each temperature measurement method was divided into interval widths of 0.3 °C each, from 35.1 to 37.2 °C, and less than 35.1 °C and greater than 37.2 °C. The skewness and kurtosis were determined for each distribution (IBM SPSS Statistics for Windows, Version 22.0., IBM Corp., NY, USA). We hypothesized that the skewness and kurtosis were both 0 if the data showed normal distribution.

The difference of means between *T*_reg_, *T*_ax-10 min_, *T*_ax-10 s_, and *T*_ty_ was assessed by the one-way analysis of variance using SPSS software. A post hoc test was conducted using the Bonferroni method.

The correlations between *T*_ax-10 min_ and *T*_ax-10 s_, *T*_ax-10 min_ and *T*_ty_, and *T*_reg_ and *T*_ax-10 s_ were evaluated by Pearson’s test. Fisher’s z-transformation test was performed to examine the difference between the correlations. Linear regression analysis was also conducted using the method of least squares.

We assumed a causal relationship between *T*_ax-10 s_ results and the questionnaire answers. First, we divided the subjects into two groups based on their *T*_ax-10 s_: one group with measurements lower than the *T*_ax-10 s_ median and the other with higher measurements. Each answer of the questionnaire was digitized and compared between the two groups using Student’s *t* test. The null hypothesis was rejected at *P* < .05. All values are expressed as the mean ± SD.

## Results

**Fig. 1:**
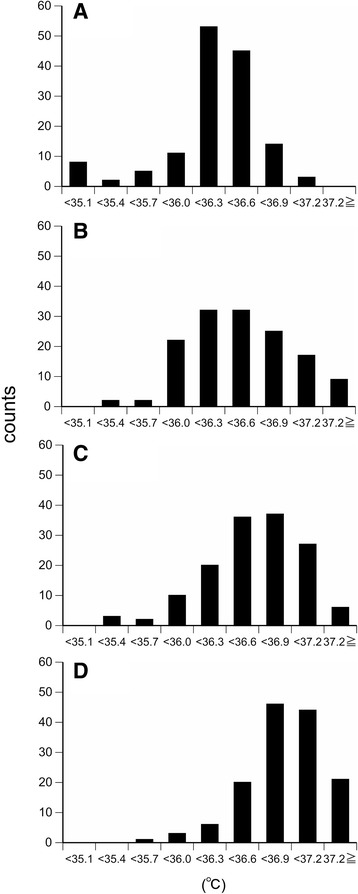
Histograms demonstrating the grouped *T*_reg_, *T*_ax-10 s_, *T*_ax-10 min_, and *T*_ty_ data. Histograms of **a** *T*_reg_ (median = 36.2 °C, skewness = −0.40, kurtosis = 0.51), **b** *T*_ax-10 s_ (median = 36.4 °C, skewness = 0.04, kurtosis = −0.28), **c** *T*_ax-10 min_ (median = 36.5 °C, skewness = −0.66, kurtosis = 0.54), and **d** *T*_ty_ (median = 36.9 °C, skewness = −0.82, kurtosis = 1.02) in healthy young men and women (*n* = 141). The data for each temperature measurement method was divided into interval widths of 0.3 °C, from 35.1 to 37.2 °C, and less than 35.1 °C and greater than 37.2 °C. *T*_reg_, the regular body temperature each subject reported in the questionnaire, *T*_ax-10 s_, axillary temperature measured with a digital thermometer in a predictive mode (10-s measurement), *T*_ax-10 min_, axillary temperature obtained using a standard method (10-min measurement), *T*_ty_, tympanic membrane temperature by infrared thermometry

Figure 1a–d shows the frequency distributions of *T*_reg_, *T*_ax-10 s_, *T*_ax-10 min_, and *T*_ty_, respectively. The mean values were 36.2 ± 0.4, 36.4 ± 0.5, 36.5 ± 0.4, and 36.8 ± 0.3 °C. Any pair of the means was different (*P* < .05). The median values of *T*_reg_, *T*_ax-10 s_, *T*_ax-10 min_, and *T*_ty_ were 36.2, 36.4, 36.5, and 36.9 °C, respectively. The skewness was −0.40, 0.04, −0.66, and −0.82 in *T*_reg_, *T*_ax-10 s_, *T*_ax-10 min_, and *T*_ty_, respectively, and the kurtosis was 0.51, −0.28, 0.54, and 1.02.

**Fig. 2:**
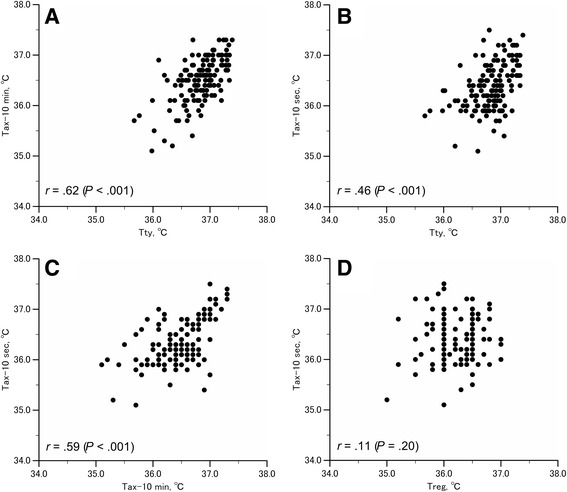
Scattergrams for *T*_ty_ and *T*_ax-10 min_, *T*_ty_ and *T*_ax-10 s_, *T*_ax-10 min_ and *T*_ax-10 s_, and *T*_reg_ and *T*_ax-10 s_. Scattergrams for *T*_ty_ and *T*_ax-10 min_ (**a**), *T*_ty_ and *T*_ax-10 s_ (**b**), *T*_ax-10 min_ and *T*_ax-10 s_ (**c**), *T*_reg_ and *T*_ax-10 s_ (**d**) from healthy young men and women (*n* = 141)

Figure 2 shows scattergrams demonstrating the relationship between *T*_ty_ and *T*_ax-10 min_ (A), *T*_ty_ and *T*_ax-10 s_ (B), *T*_ax-10 min_ and *T*_ax-10 s_ (C), and *T*_reg_ and *T*_ax-10 s_ (D). There were significant correlations between *T*_ty_ and *T*_ax-10 min_, *T*_ty_ and *T*_ax-10 s_, and *T*_ax-10 min_ and *T*_ax-10 s_ (*r* = .62, *P* < .001; *r* = .46, *P* < .001; and *r* = .59, *P* < .001, respectively). However, there was no significant correlation between *T*_reg_ and *T*_ax-10 s_ (*r* = .11, *P* = .20). The linear regression line equations for *T*_ty_ and *T*_ax-10 min_, *T*_ty_ and *T*_ax-10 s_, and *T*_ax-10 min_ and *T*_ax-10 s_ were: *y = 0.82x + 6.26*, *y = 0.64x + 12.94*, and *y = 0.62x + 13.80*, respectively. The *r*-value for *T*_ty_ and *T*_ax-10 min_ was greater than that for *T*_ty_ and *T*_ax-10 s_ (*z* = 2.64, *P* = .01).

Table 1 summarizes the comparison of each answer in the questionnaire between the two groups we had defined by subject *T*_ax-10 s_. The group with a *T*_ax-10 s_ below the median *T*_ax-10 s_ had a lower body mass index and women in the groups that had irregular menstrual cycles.

**Table 1:** The comparison of each answer in the questionnaire between the two groups defined by *T*_ax-10 s_

	Mean ± SD	*T*_ax-10 s_ < 36.4 °C	*T*_ax-10 s_ ≥ 36.4 °C	*P* value
1. Body mass index (height and body weight)	20.5 ± 2.2	20.0 ± 1.9	21.1 ± 2.4	.01*
2. How many times do you have meal each day?	2.8 ± 0.5	2.8 ± 0.4	2.9 ± 0.6	.38
3. Do you eat between meals?	1.9 ± 0.8	1.9 ± 0.8	2.0 ± 0.7	.56
Answer (1: frequently 2: sometimes 3: seldom)				
4. Do you eat after midnight?	2.3 ± 0.8	2.3 ± 0.8	2.4 ± 0.8	.81
Answer (1: frequently 2: sometimes 3: seldom)				
5. How many days do you drink alcohol a week?	1.0 ± 1.5	1.0 ± 1.4	1.0 ± 1.5	.75
6. Do you think your diet is well-balanced?	1.5 ± 0.5	1.5 ± 0.5	1.5 ± 0.5	.82
Answer (1: yes 2: no)				
7. Do you try reducing caloric intake to control body weight?	1.9 ± 0.3	1.9 ± 0.3	2.0 ± 0.2	.16
Answer (1: yes 2: no)				
8. How many hours do you sleep each night?	6.7 ± 1.2	6.7 ± 1.3	6.8 ± 1.2	.82
9. How many hours do you exercise a week?	3.9 ± 6.1	3.4 ± 5.3	4.8 ± 6.9	.19
10. Do you think that you are very sensitive to a cold environment?	1.6 ± 0.5	1.5 ± 0.5	1.7 ± 0.5	.12
Answer (1: yes 2: no)				
11. How often do you use an air conditioner in the summer?	1.9 ± 0.8	1.9 ± 0.8	2.0 ± 0.8	.51
Answer (1: frequently 2: sometimes 3: seldom)				
12. What is your normal body temperature?	36.2 ± 0.4	36.2 ± 0.4	36.2 ± 0.4	.20
13. For females only: Do you have a regular menstrual cycle?	1.3 ± 0.5	1.5 ± 0.5	1.2 ± 0.4	.01*
Answer (1: yes 2: no)				

*P* values were calculated using Student’s *t* test

*P value <.05

## Discussion

The term “normal body temperature” is often used in clinical medicine and at home; however, the definition should be more sharply defined to avoid misunderstanding. We aimed to answer three fundamental questions regarding the normal body temperature (usually assessed by the value of axillary temperature) in the present study. First, we analyzed if variation in axillary temperature between subjects originated from (a) technical errors in the measurement process, for example, incorrect placement of the measurement device, (b) technological problems with the instrument itself, for instance, with the predictive algorithm, or (c) individual differences in core body temperature. Second, we tested whether the body temperature subjects identified as their personal body temperature corresponded with their measured body temperature. Third, we investigated if differences in axillary temperature reflect differences in physical, physiological, or behavioral characteristics or vice versa.

We obtained *T*_ty_ using continuous infrared thermometry as a surrogate for estimating core temperature. It has been reported that the value obtained by this method correlates well with the esophageal temperature at an ambient temperature of 19–24 °C [25]. The ambient temperature is a factor affecting the reliability of the infrared thermometry. In addition, the sensor is needed to face to the tympanic membrane. The sensor probe used in the present study was designed to correct the influence by monitoring the ambient temperature and to fit to the ear canal, pointing to the tympanic membrane (based on the manual of the maker). We also tried to increase the reliability of the measurement by the methods as follows, besides collecting data of more than 100 subjects. Measurements were conducted in a stable thermoneutral environment to minimize deviations from the core temperature. A researcher conducted the placement of the probe, making the sensor face to the tympanic membrane. The probe occluded the ear canal, which also minimized the influence of the ambient temperature. Moreover, we continuously measure *T*_ty_, till when the value became stable (usually took 10–15 min). We assume that, even if the sensor did not correctly point to the tympanic membrane, the stabilizing period allowed the inner-ear temperature to become identical to the temperature of the tympanic membrane. The average was 36.8 ± 0.3 °C, and the coefficient was 0.8%. This finding together with the higher value of the kurtosis for the frequency distribution of *T*_ty_ could suggest that there was little interindividual difference in the core temperature. Although the skewness was less than 0, the result may also suggest the accuracy of the measurement method (Fig. 1d).

Reports indicate that axillary temperature, although measured at the body surface, correlates well with the core temperature [11, 13, 17, 21–24]. In the present study, we also found a significant correlation between *T*_ty_ and *T*_ax-10 min_ (Fig. 2a), although the frequency distribution of *T*_ax-10 min_ was different from that of *T*_ty_ (smaller kurtosis, Fig. 1c, d)_._ Moreover, the regression slope was 0.82. These results may suggest that under the conditions present during our measurements, axillary temperature closely approximates the core temperature as previously reported. However, the value showed greater variation among subjects compared to *T*_ty_. In addition, *T*_ty_ was higher than *T*_ax-10 min_ as previously reported [28]. Because the measurements were conducted in a similar environment and under the instruction and supervision of researchers, factors leading to measurement errors [9] may have been negligible.

There was a significant correlation between *T*_ty_ and *T*_ax-10 min_, and *T*_ty_ and *T*_ax-10 s_ (Fig. 2a, b). The correlation coefficient (*r*) for the correlation between *T*_ty_ and *T*_ax-10 s_ was lower than that for the correlation between *T*_ty_ and *T*_ax-10 min_. These results confirm that *T*_ax-10 s_ includes a greater error in estimated axillary temperatures as indicated by previous reports [12–14]. Moreover, the mean of *T*_ax-10 s_ was lower than that of *T*_ax-10 min_, which suggests that it is necessary to know an individual’s personal regular temperature as obtained by the standard method. This knowledge may help individuals to assess whether they have a fever correctly and, thus, evaluate their state of health more accurately.

It seems to be accepted that “normal body temperature” is around 37.0 °C on average, although a range around this value (36.2 to 37.5 °C) is considered within normal limits [15, 16]. However, in the present study, the averaged values estimated by axillary temperature measurements using a digital thermometer were lower than the accepted normal value (i.e., 36.2 °C on average). The reason remains unclear. Differences in sensor material and the use of a predictive mode may have resulted in lower values.

In recent years, the axillary temperature is usually measured using a predictive mode, and all of the study participants regularly used this method of measurement. However, we did not find any correlation between *T*_reg_ and *T*_ax-10 s_ (Fig. 2d). This result may suggest that the body temperature subjects believe to be their regular temperature is not based on the values they previously measured. However, we may have misinterpreted the data, because axillary temperature shows daily fluctuations and is influenced by the menstrual cycle [29, 30].

Subjects with a *T*_ax-10 s_ below the median tended to have a lower body mass index (28% underweight, 0% overweight) compared to those whose *T*_ax-10 s_ was above the median (14% underweight, 78% normal weight, 8% overweight) (Table 1). A smaller subcutaneous fat mass may affect axillary temperature and be a factor involved in the interindividual difference we found. In addition, female subjects in the former group had irregular menstrual cycles. The disturbance of body temperature related to irregular menstrual cycles may influence axillary temperature.

Twenty subjects (14% of the total subjects) reported a *T*_reg_ below 36.0 °C. Eight of these subjects had a *T*_ax-10 s_ of <36.0 °C. Although we did not find any relationship between these two variables (Fig. 2d) for all subjects, these eight subjects may have reported their regular body temperature based on the knowledge of previous actual measurements.

An individual’s axillary temperature is judged as normal or abnormal based on a previously determined value (i.e., 37.0 °C), and it is well known that normal body temperature varies [15, 16]. The present results may indicate that the normal temperature range has changed due to the use of digital thermometers that utilize predictive algorithms. Our experiment included young and healthy subjects in an environment controlled to minimize factors that could potentially affect axillary temperature; although, we did not consider gender differences including those related to menstruation. Even in the experimental environment, we found important interindividual differences. The present study suggests that each individual needs to be aware of his or her baseline temperature. The interindividual differences in axillary temperature may, in part, reflect measurement errors. However, we found a lower *T*_ax-10 s_ that was associated with lower body mass indices in male and female subjects and with irregular menstrual cycles. Because we did not assess physiological parameters such as metabolism, skin temperature, and skin blood flow, which may influence axillary temperature, the reasons for the observed interindividual variation remain unclear. Future studies are needed to clarify the mechanism underlying this variation.

## Conclusions

Modern axillary temperature measurement techniques may have changed the range of normal body temperature; nonetheless, they are reliable enough to estimate the core body temperature adequately. However, even between young and healthy subjects at rest in a comfortable environment, a normal temperature shows significant variation. Our results suggest that each individual should know his or her own regular temperature. Moreover, axillary temperature may reflect individual physical differences and diverse physiological states. Axillary temperature still has importance in evaluating health status, not only for determining if a fever is present but also for defining our baseline state of health.

## Abbreviations

*T*_ax-10 min_: Axillary temperature using the standard mode, taking 10 min
*T*_ax-10 s_: Axillary temperature using the predictive mode, taking 10 s
*T*_reg_: Regular body temperature
*T*_ty_: Tympanic membrane temperature
